# Developmental Biology: Computational and Experimental Approaches—2nd Edition

**DOI:** 10.3390/ijms27031461

**Published:** 2026-02-01

**Authors:** Mikhail Ponomarenko

**Affiliations:** Institute of Cytology and Genetics, Siberian Branch of the Russian Academy of Sciences (ICG SB RAS), 630090 Novosibirsk, Russia; pon@bionet.nsc.ru

Developmental biology is focused on the study of all phenotypic traits responsible for the timely and adequate progression of all molecular genetic, biochemical, and morphogenetic processes as the norm of the corresponding biological species in an individual living organism (ontogenesis) from either fertilization during sexual reproduction or separation from the maternal organism during asexual reproduction to the end of its life [1]. Both computational and experimental approaches within this field of intense research rest on empirical information about molecular mechanisms of gene expression and signal transduction. The mechanisms form the basic level in the hierarchy from intracellular processes through multicellular self-organization in tissues and the individual organism’s formation to its interaction with the environment, including other individuals of various species [2]. Among the most recent outstanding achievements on this matter, we should emphasize the identification of differences between developmental genes and all other gene types, mainly housekeeping, genes in the context of chromatin packaging, intron–exon structure, promoter organization, transcription factors regulating gene expression, and both the timing and sites of DNA replication initiation [3].

When the World Health Organization declared 2020–2030 the Decade of Healthy Aging, the extension of lifespan and life quality improvements became the mainstream of experimental and computational research in developmental biology. Attention was focused on the efficiency of treatments for age-related diseases, such as dementia and cancer, via side effect prevention, wound healing improvement, post-traumatic recovery, tissue regeneration using induced pluripotent stem cells, assisted reproductive technologies, and epigenetic reprogramming [4].

In 2025, the following contributions were included in our Special Issue of the International Journal of Molecular Sciences, entitled “Developmental Biology: Computational and Experimental Approaches—2nd Edition”:

- Two biomedical research articles (Contributions 1 and 2) on mouse and rat animal models of human developmental diseases, respectively;
- One computational research article (Contribution 3);
- One surprising article (Contribution. 4) of the extremely rare intermediate type “Research in context” introduced by an editorial comment by the *Lancet* [5], whose standardized label is “Review”, because it combines both the computational analysis performed by its authors and their comprehensive review of the literature on the subject;
- One classic review article (Contribution 5).

The research article by Tkachenko et al. (Contribution 1) presents the potential side effects of the widely used antidepressant fluoxetine, one of the best drugs for treating motor delays, excessive daytime sleepiness, or excessive sleep duration, on the reproductive health of women and their offspring. They used female outbred ICR (Institute of Cancer Research) mice bred to achieve superior reproductive and maternal performance, including excellent litter quality. Although fluoxetine impaired oogenesis by disrupting cytoplasmic maturation, the litter sizes and ovarian reserves of the offspring remained unchanged in fluoxetine-treated females compared to the norm, whereas the fluoxetine-related offspring unexpectedly showed an increase in body mass, which motivated an in-depth study of the age dependence of susceptibility to the side effects of fluoxetine. As a result, the greatest vulnerability of adolescent ovaries was revealed [6].

In another biomedical research paper (Contribution 2), Demyashkin and co-authors investigated a biomedical model for the prevention of radiation-induced pancreatitis as a side effect of modern radio-electronic anticancer therapy. The model involved 8–9-week-old male Wistar rats treated with N-acetylcysteine as an adjuvant prophylactic medication with radioprotective antioxidant properties. This work continued their intensive pharmacological search among well-studied molecular medications with long histories of implementation in the treatment of a wide range of diseases. An additional objective was to improve the effectiveness and mitigate the side effects of radiation oncology, thereby accelerating and economizing the development of such drugs (e.g., [7]).

Ivanov and co-authors (Contribution 3) exemplified how in silico computational approaches within genome-wide omics meta-analysis can expand our knowledge of developmental biology by integrating the multitude of independent experimental datasets, such as clinical transcriptomic profiling of patients at different stages of cancer [8]. By now, the authors have summarized and extended their ongoing efforts to apply phylostratigraphic reasoning—i.e., the association of genes with phylostratigraphic age indices (PAIs) based on orthology—to the interpretation of molecular mechanisms of human disease emergence and development. This line of work started with their analysis of KEGG Human Diseases gene networks using Orthoscape [9], where Mustafin et al. [10] formalized how PAIs can be combined with measures of genetic variability and selection regime in disease-associated networks. The authors reported that (1) most genes in disease networks are consistent with stabilizing selection, (2) younger genes tend to be more variable, and (3)—in the oncology context—that gene networks associated with specific cancer types are significantly enriched in evolutionarily ancient genes, which supports the view that malignancy preferentially engages deeply conserved cellular programs. Resting on Orthoscape’s conceptual and analytical basis, Ivanov et al. [11] introduced Orthoweb (version 1.0.0), a software package created for the evolutionary analysis of both individual genes and gene networks. It enables the calculation of PAIs and divergence (DI) metrics, as well as transcriptome-scale measures, such as the Transcriptome Age Index (TAI) and Transcriptome Divergence Index (TDI).

Next, the authors employed both the Orthoscape [9] and Orthoweb [11] toolboxes to investigate a wide range of academic and practical problems in developmental biology: the investigation of abiotic stress in plants [12], and appetite [13], thermoregulation [14], cholesterol biosynthesis [15], the renin–angiotensin–aldosterone system (RAAS) [16], pain generation, perception, response, and anesthesia [17] in animals; the creation of the HH_Signal_pathway_db knowledge base on the hedgehog signaling pathway during human development [18] and the GlucoGenes^®^ knowledge base on glucose metabolism disorders in humans [19]; and the comparison of gene networks in autism spectrum disorders and Alzheimer’s disease in humans [20].

The authors of Contribution 3 used the mentioned multifaceted studies of the computational toolboxes in question as grounds for the research of both the emergence and development of cancer by means of examining TAI across four pathological stages in numerous tumor types. They made it clear that carcinomas generally show a lower TAI than corresponding normal tissues and identified a statistically supported “hourglass” pattern in breast ductal carcinoma, bladder urothelial carcinoma, and hepatocellular carcinoma, where early and late stages exhibit higher a TAI (the greater impact of evolutionarily newer genes), whereas intermediate stages show a dip (an increased impact of evolutionarily older genes). This pattern is consistent with a conserved evolutionary trajectory during tumor progression. This may thus unexpectedly correspond to Karl von Baer’s third law for the development of multicellular organisms in terms of their molecular evolution [21].

Contribution 4 was a review by Koveshnikova and Kuznetsova that summarizes publications and databases on the human *AICDA* gene, a key player in adaptive immunity, antibody diversification, and epigenetic regulation. The authors considered all single-nucleotide polymorphisms (SNPs) of this gene according to the NCBI dbSNP database [22]. They additionally characterized each SNP with the results of their own computational analysis using publicly available bioinformatics web services.

Contribution 5 by Davletgildeeva and Kuznetsov concludes this Special Issue. It reviews in detail the molecular mechanisms of R-loop formation and its function in transcription–replication conflicts (TRCs). Such conflicts are a risk factor for DNA damage and genomic instability, both able to cause cell death or carcinogenesis, which are key events in developmental biology. Eventually, this review forms the basis for an in vitro biochemical experiment, allowing for a thorough step-by-step characterization of the functioning of all types of molecules during head-on TRCs [23].

Our Special Issue on computational and experimental approaches in developmental biology illustrates the key role played by the elucidation of molecular mechanisms of signal transduction, gene expression, and genome functioning for use in healthcare (Contribution 1), pharmacology (Contribution 2), disease prevention (Contribution 3), adaptive immunity improvement (Contribution 4), resilience to external and internal stressors (Contribution 5), and, in the long run, healthy longevity and lifespan prolongation.

## Abbreviations

DI	Divergence Index
PAI	Phylostratigraphic Age Index
RAAS	renin–angiotensin–aldosterone system
SNP	single-nucleotide polymorphism
TAI	Transcriptome Age Index
TDI	Transcriptome Divergence Index
TRC	transcription-–replication conflict
